# Differentiation of keratinocytes or exposure to type 2 cytokines diminishes *S. aureus* internalization

**DOI:** 10.1128/msphere.00685-23

**Published:** 2024-03-19

**Authors:** Alexis R. Morgenstern, Liam F. Peterson, Kimberly A. Arnold, Matthew G. Brewer

**Affiliations:** 1 Department of Dermatology, University of Rochester, Rochester, New York, USA; 2 Department of Pathology & Laboratory Medicine, University of Rochester, Rochester, New York, USA; University of Nebraska Medical Center College of Medicine, Omaha, Nebraska, USA

**Keywords:** keratinocyte, *S. aureus*, inflammation, bacterial invasion

## Abstract

*Staphylococcus aureus* is a leading cause of skin and soft tissue infections. Colonization by this bacterium is increased in individuals with chronic cutaneous diseases such as atopic dermatitis, psoriasis, and bullous pemphigoid. The greater abundance of *S. aureus* on the skin of subjects with atopic dermatitis in particular has been linked to recurrent cutaneous infections. The primary cell type of the epidermal layer of the skin is the keratinocyte, and it is thought that *S. aureus* internalized in keratinocytes associates with an increased incidence of skin infections. This study addresses whether keratinocyte differentiation and/or inflammation, two important characteristics altered in cutaneous diseases, influence bacterial internalization. To do this, *S. aureus* internalization was measured in immortalized and primary keratinocytes that were differentiated using high Ca^2+^-containing media and/or exposed to cytokines characteristic of atopic dermatitis (IL-4 and IL-13) or psoriasis (IL-17A and IL-22) skin. Our results indicate that *S. aureus* internalization is uniquely decreased upon keratinocyte differentiation, since this was not observed with another skin-resident bacterium, *S. epidermidis*. Additionally, treatment with IL-4 + IL-13 diminished bacterial internalization. We interpret this decrease as a mechanism of keratinocyte-based bacterial killing since a similar number of bacterial genomes were detected in cytokine-treated cells, but less viable internalized *S. aureus* was recovered. Finally, of the receptors reported for *S. aureus* binding/internalizing into keratinocytes, expression of the α_5_ component of the α_5_β_1_ integrin was in greatest accordance with the number of internalized bacteria in the context of keratinocyte differentiation.

**IMPORTANCE:**

Individuals with chronic cutaneous diseases demonstrate heightened susceptibility for severe and recurrent infections from *Staphylococcus aureus*. What drives this altered susceptibility remains poorly understood. Previous publications have detected *S. aureus* as deep as the dermal layer of skin in subjects with atopic dermatitis, suggesting that the cutaneous environment of this disease enables deeper bacterial infiltration than occurs in healthy individuals. This observation indicates that *S. aureus* has greater opportunity to interact with multiple skin cell types in individuals with chronic inflammatory skin diseases. Identifying the characteristics of the skin that influence bacterial internalization, a common method to establish reservoirs and evade the immune response, is critical for our understanding of *S. aureus* pathogenesis. The significance of this research is the novel identification of epidermal characteristics that influence *S. aureus* internalization. With this knowledge, methods can be developed to identify patient populations at greater risk for cutaneous infections.

## INTRODUCTION

A common struggle for individuals with chronic cutaneous diseases arises from recurrent skin colonization and infection by the bacterium *Staphylococcus aureus* (*S. aureus*) (1–3). In addition to infections, this bacterium has been shown to exacerbate atopic dermatitis (AD) disease severity and a recent study suggested it contributes (either onset and/or severity) to bullous pemphigoid (4–6). One hypothesis to explain this occurrence is that *S. aureus* internalizes and persists more efficiently in the main epidermal cell known as the keratinocyte, providing a long-term reservoir for colonization and infection. Since bacterial internalization is a common method to escape immune clearance and antibiotic destruction (7, 8), it is possible that in subjects with chronic skin diseases this occurs more frequently. Therefore, identifying characteristics that influence *S. aureus* internalization into keratinocytes is important for understanding infection susceptibility and pathogenesis in individuals with chronic cutaneous diseases.

A defining characteristic of chronic cutaneous diseases, such as AD or psoriasis, is a unique cytokine milieu within the skin. In subjects with AD, this is predominantly observed by overexpression of type 2 inflammatory mediators IL-4 and IL-13 (9–12). Notably, the cutaneous cytokine profile can vary across AD subjects and is most likely influenced by genetic and environmental factors. For example, African Americans with AD have increased expression of the type 3 cytokine IL-22 within the skin in addition to type 2 cytokines (13). In contrast, individuals with psoriasis demonstrate a dominant type 3 cytokine profile in the skin with marked expression of IL-17A and IL-22 (14, 15). Differential expression of cytokines in the skin of subjects with chronic cutaneous diseases may contribute to their susceptibility for persistent pathogen colonization.

An important feature of the skin is the capacity of keratinocytes to differentiate and form the multilayered epidermis (16, 17). This provides various layers that have fundamentally different biology, including the stratum basale, stratum spinosum, stratum granulosum, and stratum corneum. The stratum basale is a single layer of proliferative keratinocytes at the bottom of the epidermis that provides the cells necessary to seed the upper layers of the epidermis (18). Adjacent to the stratum basale is the multi-layered stratum spinosum, which is where the transition to fully differentiated, skin barrier-forming keratinocytes occurs (18). The second to last layer of the epidermis is the stratum granulosum. This epidermal layer is composed of three cell layers and provides the second barrier of the skin known as tight junctions (19). Finally, the outermost layer is called the stratum corneum and is composed of dead anucleate keratinocytes, is many layers thick (influenced by anatomical location), and provides the first barrier to the body through formation of an impermeable wall (20). This epidermal “wall” is composed of bricks (dead keratinocytes) and mortar (lipids/extracellular matrix) produced as keratinocytes differentiate in the skin (21). While *S. aureus* is most often detected on the stratum corneum, in subjects with AD, which are known for their disrupted epidermal barrier function (22, 23), *S. aureus* has been detected as low as the dermal layer of the skin (24, 25). These observations indicate that in chronic cutaneous diseases, all keratinocyte differentiation states can be exposed to this bacterium, but whether keratinocyte differentiation state influences *S. aureus* internalization has not been investigated.

Since AD subjects are well characterized to have recurrent infections and elevated colonization by *S. aureus*, we hypothesized that treatment of keratinocytes with the canonical AD-relevant cytokines, IL-4 + IL-13, promotes bacterial internalization. To identify whether differentiation and/or specific cytokine milieus observed in chronic cutaneous diseases influences bacterial internalization, this study utilized undifferentiated and calcium-induced differentiated keratinocytes treated with IL-4 + IL-13, IL-22, or IL-17A.

## RESULTS

**Fig 1:**
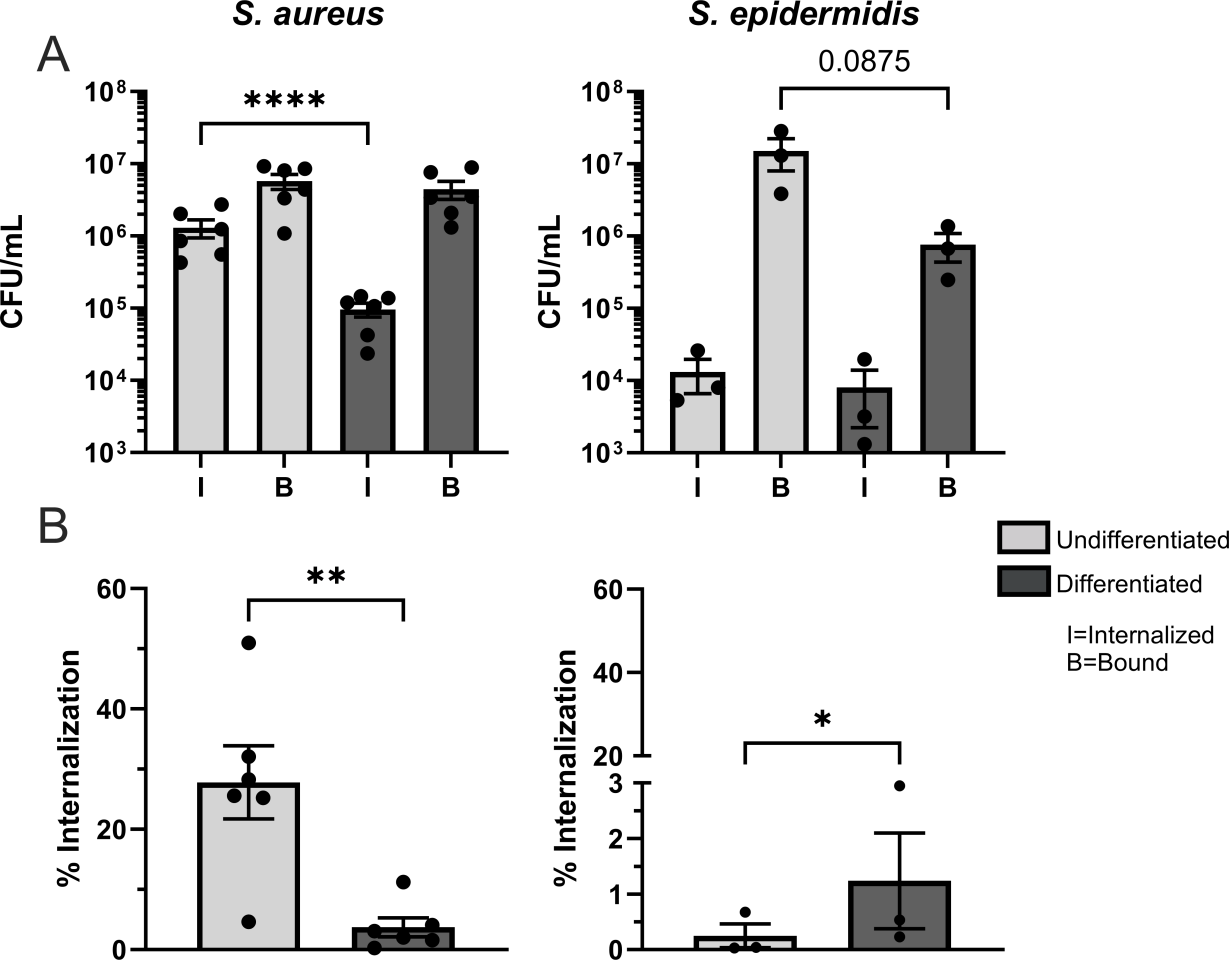
*S. aureus,* but not *S. epidermidis,* internalization is diminished upon keratinocyte differentiation. Undifferentiated and differentiated N/TERT-2G were exposed to *S. aureus* or *S. epidermidis* at a multiplicity of infection (MOI) of 100 colony-forming units (CFU) per cell for 3 h. Extracellular bacteria were killed by gentamicin treatment and cells were lysed to quantify the number of internalized bacteria. Binding was measured by subtracting the number of internalized bacteria from the total number quantified (no gentamicin). Bacterial internalization and binding are represented by CFU/mL (**A**) and the percent internalization (internalized divided by bound, (**B**) of *S. aureus* and *S. epidermidis* at different stages of keratinocyte differentiation. Data are shown as mean ± SEM. Statistical significance was tested with a paired *t* test, *n* = 3 (*S. epidermidis*) or 6 (*S. aureus*) experiments. * *P* < 0.05, ** *P* < 0.01, **** *P* < 0.0001.

A key feature of the epidermis is the ability to stratify into multiple layers, which is due to the differentiation of keratinocytes. We utilized a standard method in the field to induce keratinocyte differentiation *in vitro*, which is through exposure to media containing a high concentration of calcium (1.3–1.8 mM) (22, 26–28). This method was chosen for these studies because of the key role calcium ions play in the formation of the epidermis (29). We first determined the impact of keratinocyte differentiation on *S. aureus* internalization. The number of viable internalized bacteria was measured from undifferentiated and differentiated (24 h post-exposure to high calcium media) keratinocytes, through a gentamicin-based binding/internalization assay. To identify optimal experimental parameters for this assay in keratinocytes, different amounts of bacteria were used to determine the ideal multiplicity of infection (MOI). An MOI of 100 was chosen for all subsequent assays because it provided substantial numbers of internalized *S. aureus* while avoiding keratinocyte death, which we interpreted by the drop in internalized bacteria at the MOI of 500 condition (Fig. S1). Additionally, we confirmed that gentamicin was not a confounding factor within this study with an LDH release assay, which showed there was no impact on N/TERT-2G cell viability after exposure to this antibiotic (Fig. S2). After establishing the parameters for the keratinocyte binding/internalization assay, we observed that *S. aureus* internalization was significantly diminished upon keratinocyte differentiation when compared to undifferentiated samples (Fig. 1). Importantly, the number of bacteria bound to the surface of either undifferentiated or differentiated keratinocytes remained unchanged, suggesting that changes to internalization were not due to decreased bacterial binding. To test whether our observations were unique to *S. aureus* we utilized another skin-resident bacterium (*S. epidermidis*) in our binding/internalization assays. *S. epidermidis* was able to robustly bind to keratinocytes, but did not undergo substantial internalization (Fig. 1). This was noted by a 1–2 log reduction in *S. epidermidis* internalization compared to *S. aureus* (Fig. 1). Unlike *S. aureus, S. epidermidis* binding to keratinocytes diminished with differentiation, which drove the significant increase in percent internalization (Fig. 1B) observed with this bacterium. This suggests that *S. aureus* is more efficient at being internalized into keratinocytes compared to another bacterium commonly found on the skin.

**Fig 2:**
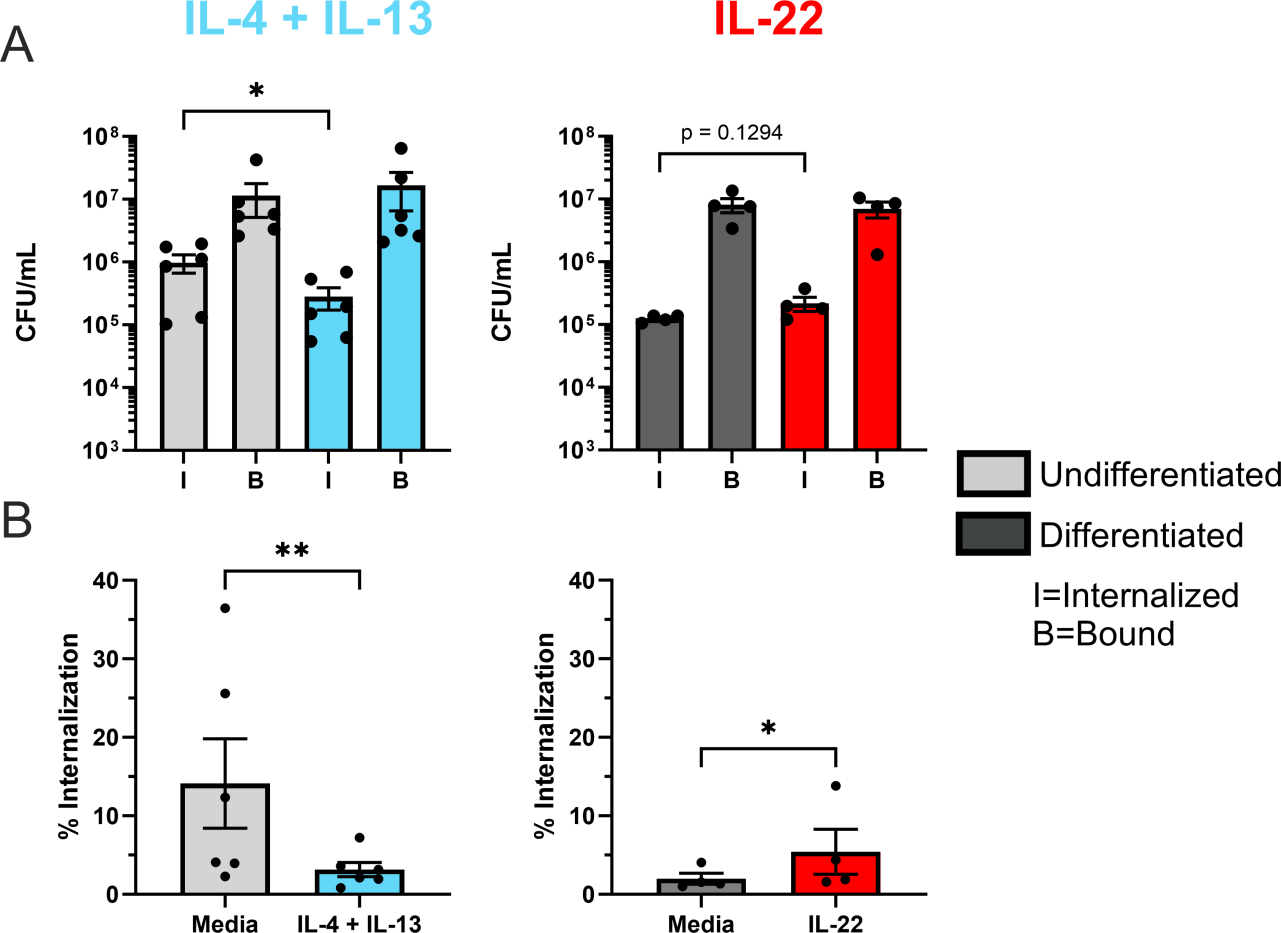
*S. aureus* internalization is decreased in undifferentiated keratinocytes treated with IL-4 + IL-13 and increased in IL-22-treated differentiated keratinocytes. Undifferentiated and differentiated N/TERT-2G were treated with either IL-4 + IL-13 or IL-22 at 50 ng/mL, respectively, and exposed to an MOI of 100 *S*. *aureus* CFU per cell for 3 h. Extracellular bacteria were killed via gentamicin treatment and cells were lysed to quantify the number of internalized bacteria. Binding was measured by subtracting the number of internalized bacteria from the total number quantified (no gentamicin). Bacterial internalization and binding in untreated or cytokine (IL-4 + IL-13 or IL-22) treated keratinocytes were measured in CFU/mL (**A**) and the percent internalization [internalized divided by bound, (**B**)] of *S. aureus* at different stages of keratinocyte differentiation. Data are shown as mean ± SEM. Statistical significance was tested with a paired *t* test. *n* = 4 (IL-22) or 6 (IL-4 + IL-13) experiments. *P < 0.05, **P < 0.01.

To understand the influence of inflammation observed in different chronic skin diseases on bacterial internalization, keratinocytes (either undifferentiated or differentiated for 24 h) were treated with IL-4 + IL-13, IL-22, or IL-17A and then *S. aureus* binding and internalization was assessed. IL-4 + IL-13 were utilized together in these assays because they are both implicated in AD and signal through a common receptor pairing present on the surface of keratinocytes (IL-4Rα/IL-13Rα1) (11, 12, 30). No significant difference was observed in bacterial internalization for undifferentiated keratinocytes treated with either IL-22 or IL-17A or differentiated keratinocytes treated with IL-4 + IL-13 or IL-17A (Fig. S3). Multiple concentrations were then tested for each cytokine used in the bacterial internalization assay to determine whether the phenotypes observed were dose-specific (Fig. S4). We observed a reproducible decrease in bacterial internalization across all doses of IL-4 + IL-13 tested as well as an increase in bacterial internalization in cells treated with all doses of IL-22. There was a slight increase in *S. aureus* internalization at the highest dose of IL-17A, but this was variable across experiments. Subsequent assays for IL-4 + IL-13 and IL-22 treatment focused on a dose of 50 ng/mL. Undifferentiated keratinocytes treated with IL-4 + IL-13 were observed to have significantly less (*P* < 0.05) internalized *S. aureus* (Fig. 2). Conversely, IL-22 [a cytokine present in the skin of subjects with psoriasis (14)] treatment of differentiated keratinocytes demonstrated a significant increase in *S. aureus* internalization when corrected for binding values (% internalization, Fig. 2B). Importantly, none of the cytokine treatments used in these studies altered *S. aureus* binding (Fig. 2, not shown for IL-17A). These results indicate that the cytokine milieu of the epidermis can either enhance or diminish *S. aureus* internalization.

**Fig 3:**
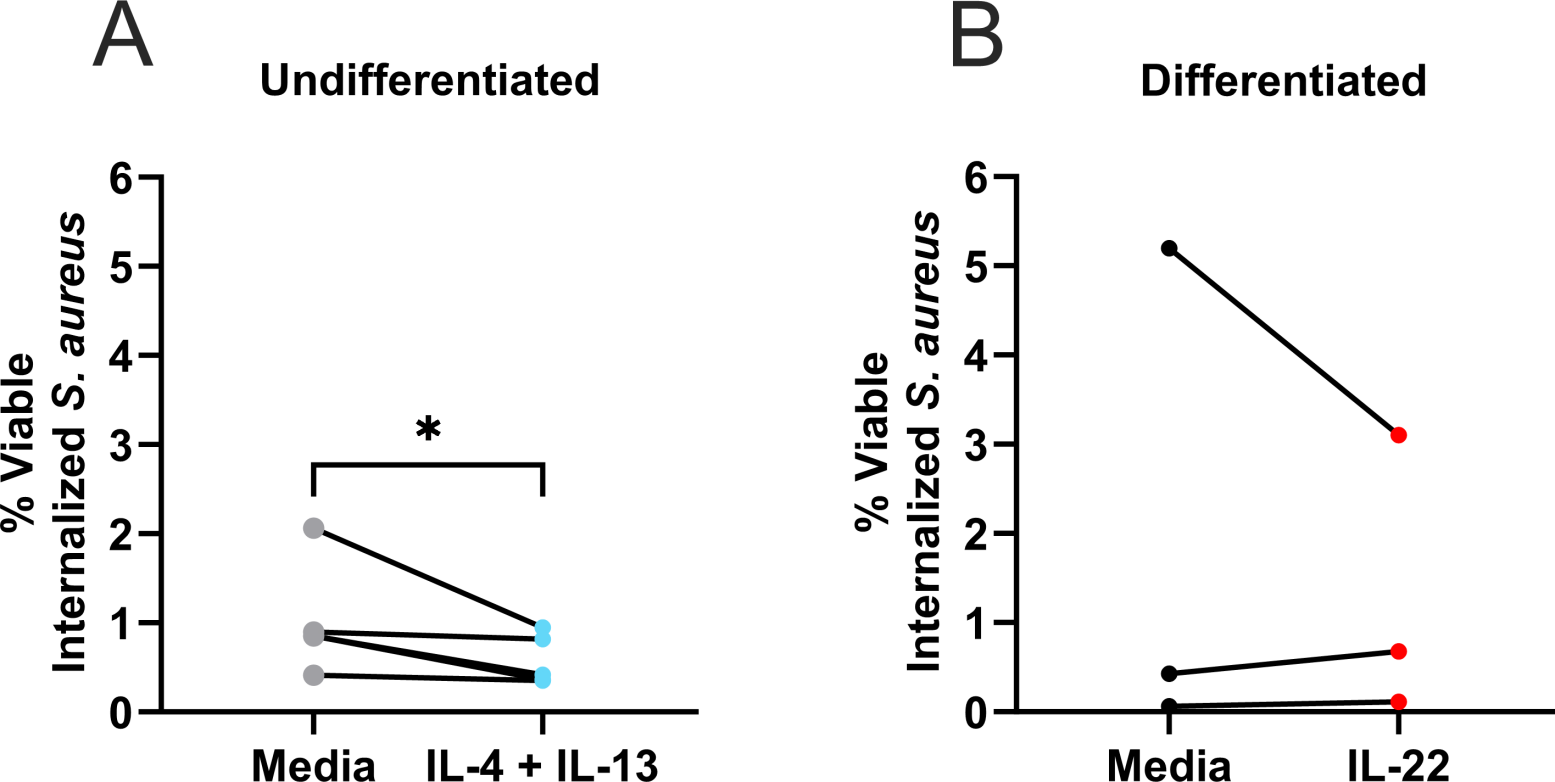
Fewer viable *S. aureus* is detected within IL-4 + IL-13-treated keratinocytes. gDNA was harvested from keratinocytes with internalized *S. aureus* (similar to Fig. 2) and the total amount of *S. aureus* in each sample was quantified through qPCR for the *femA* gene using a standard curve derived from a known number of bacteria. Results are expressed as percent viable internalized *S. aureus*, which is a measurement of the fraction of live bacteria (culturable) to the total number of bacteria (live and dead measured from qPCR). (**A**) Compares percent viability between undifferentiated keratinocytes either left untreated or exposed to IL-4 + IL-13 and (**B**) compares differentiated keratinocytes either left untreated or exposed to IL-22. Data are shown as mean ± SEM. *n* = 3 (IL-22) or 5 (IL-4 + IL-13) experiments. Each line connects an individual experiment. *P < 0.05.

A potential explanation of the differences observed in *S. aureus* internalization as a result of cytokine treatment could be attributed to killing of internalized bacteria, which has been shown to occur in keratinocytes (31, 32). To assess whether cytokine exposure influenced keratinocyte-mediated bacterial killing, we measured the viability of internalized *S. aureus*. Bacterial genomic DNA (gDNA) from internalization samples was used to quantify the amount of *femA* (a gene specific to methicillin resistance present in *S. aureus*) present (33). The number of viable *S. aureus* bacteria from internalization conditions [cultured on tryptic soy agar (TSA)] was compared to the total number of genomes from qPCR (Fig. S5A) to determine the percent viable bacteria within samples. Using this ratio (alive/total), we determined that fewer viable *S. aureus* bacteria were detected within IL-4 + IL-13-treated keratinocytes (Fig. 3A). There was no change in viable internalized bacteria detected in IL-22-treated keratinocytes (Fig. 3B), indicating an alternative explanation for the increased numbers of *S. aureus* detected within cells treated with this cytokine.

**Fig 4:**
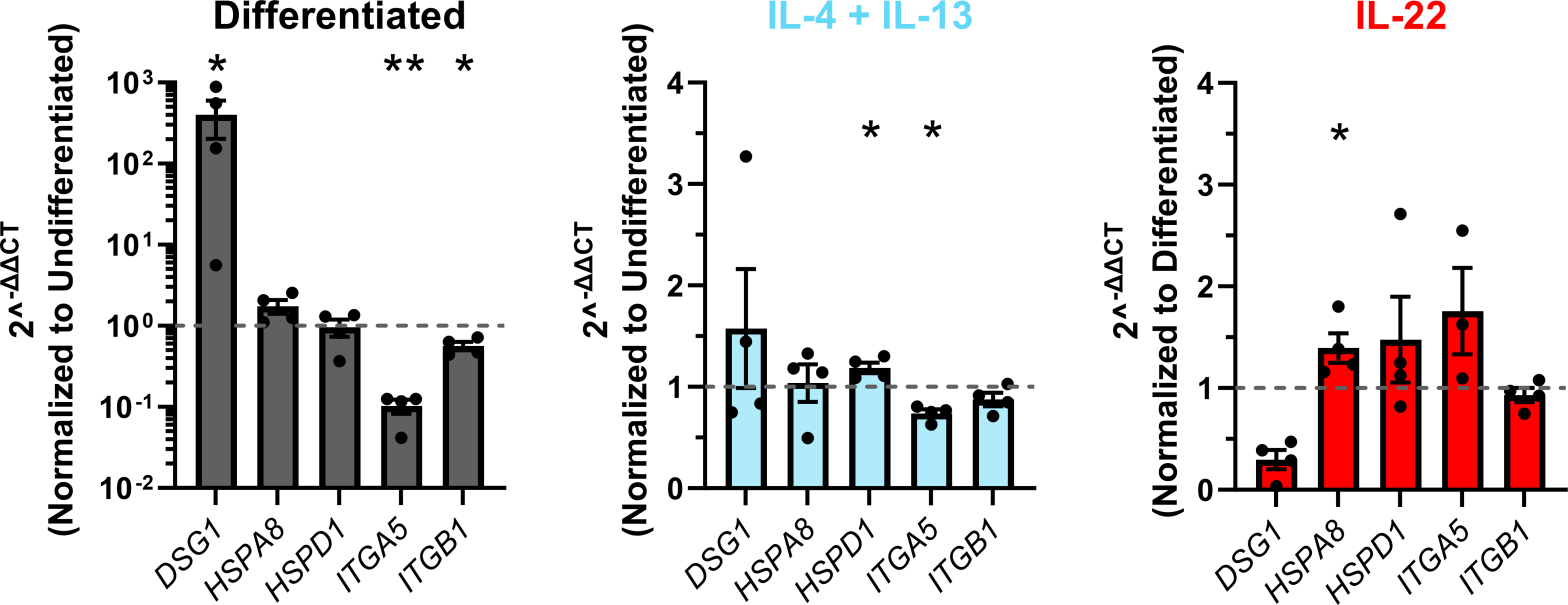
Reported *S. aureus* binding receptors have altered expression on keratinocytes undergoing differentiation or treated with cytokines. Undifferentiated and differentiated keratinocytes were treated with either IL-4 + IL-13 or IL-22 at 50 ng/mL, respectively, and mRNA expression of reported *S. aureus* binding receptors was measured by qPCR. Gene expression was normalized across samples to the level of expression for the housekeeping gene (GAPDH) of the same sample. Changes in gene expression are shown as 2^−ΔΔC*t*^, which represents the difference in expression level of the gene of interest between the treatment (differentiation or cytokine stimulation) and the control sample (either untreated undifferentiated or untreated differentiated cells). Data are shown as mean ± SEM. Statistical significance was tested with a paired *t* test. *n* = 4 experiments. *P < 0.05, **P < 0.01. The *P* value for DSG1 in IL-22-treated cells was *P* = 0.0799. An outlier was removed from the analysis of differentiated IL-22-treated keratinocytes for *ITGA5* (value 130.7).

**Fig 5:**
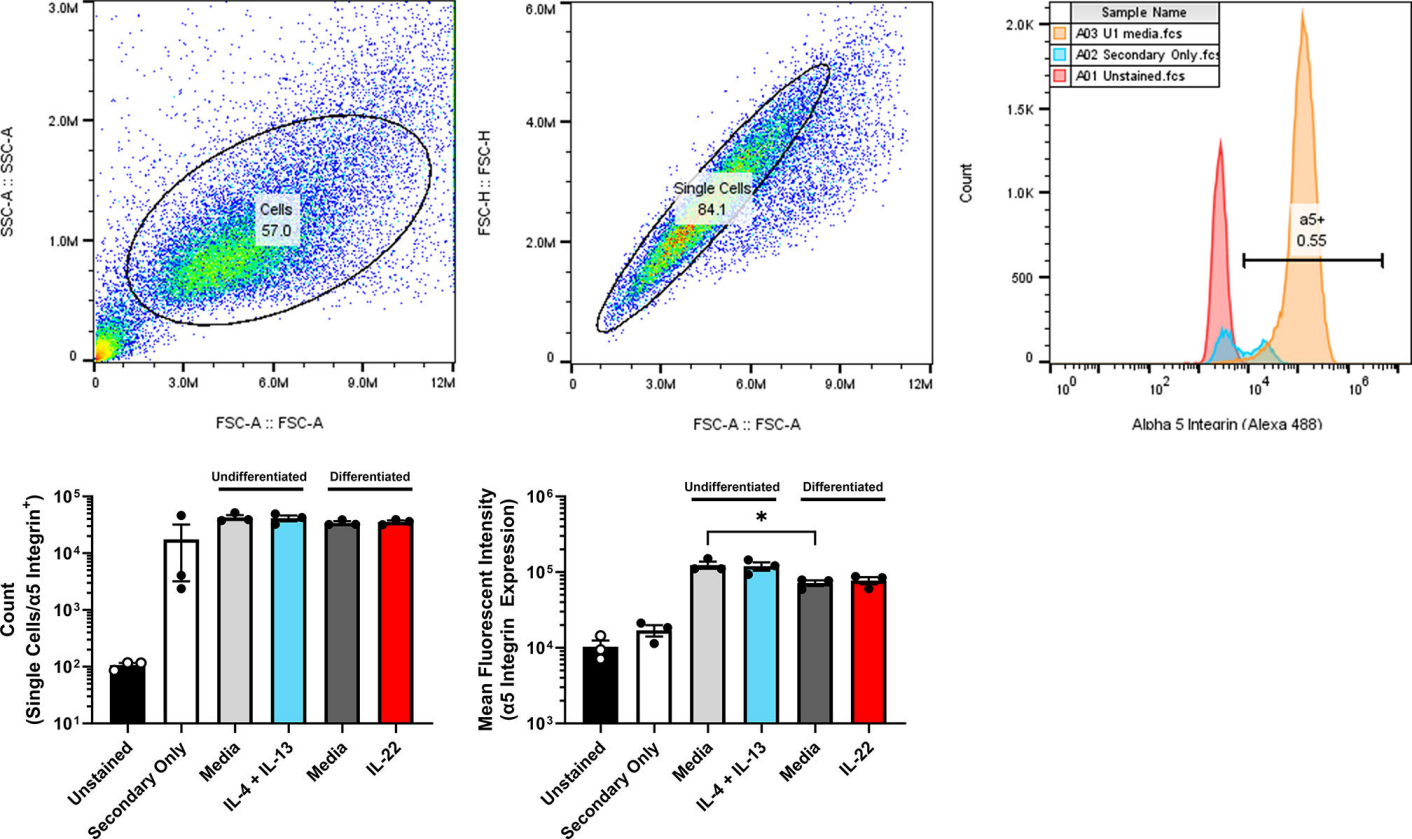
Keratinocyte surface expression of the α_5_ integrin protein is decreased upon differentiation. The top set of panels shows the gating scheme used to quantify the mean fluorescence intensity of the α_5_ integrin in undifferentiated and differentiated N/TERT-2G cells ± cytokine treatment. The gate shown in the final panel represents the percentage of α_5_ integrin-positive cells in the unstained control (red histogram). Cell count and the mean fluorescence intensity from α_5_ + cells are shown using mean ± SEM. Statistical significance was tested with a paired *t* test. *n* = 3 experiments. *P < 0.05.

We next tested whether cytokine-associated changes in bacterial internalization concur with expression of reported host cell surface receptors for *S. aureus* through qPCR analysis. Keratinocytes were either left untreated or treated with IL-4 + IL-13 or IL-22 and assessed at the relevant differentiation stages where changes were observed in bacterial internalization. Expression of the following genes: *DSG1*, *HSPA8*, *HSPD1*, *ITGA5*, and *ITGB1* was quantified since these have been indicated to interact with *S. aureus* and reported to be expressed by keratinocytes (34–37). A significant decrease in mRNA expression of *ITGA5* was observed upon keratinocyte differentiation and in undifferentiated keratinocytes treated with IL-4 + IL-13 (Fig. 4). There was also a significant increase in expression of *DSG1* in differentiated compared to undifferentiated keratinocytes (Fig. 4). We detected other significant changes in the investigated transcripts, but these were modest (less than twofold). While there were changes in gene expression of multiple binding receptors, the gene that showed the expression pattern in agreement with bacterial internalization experiments was *ITGA5*, i.e., decreased *S. aureus* internalization occurring in cells expressing less *ITGA5*. To extend the observed changes in transcript levels of *ITGA5*, we next investigated whether the same trend was observed at the protein level. Flow cytometric analysis of α_5_ integrin expression revealed a significant decrease in mean fluorescent intensity of α_5_ upon differentiation (Fig. 5). The concordant expression pattern of *ITGA5* suggests a potential role in *S. aureus* internalization as a function of keratinocyte differentiation.

**Fig 6:**
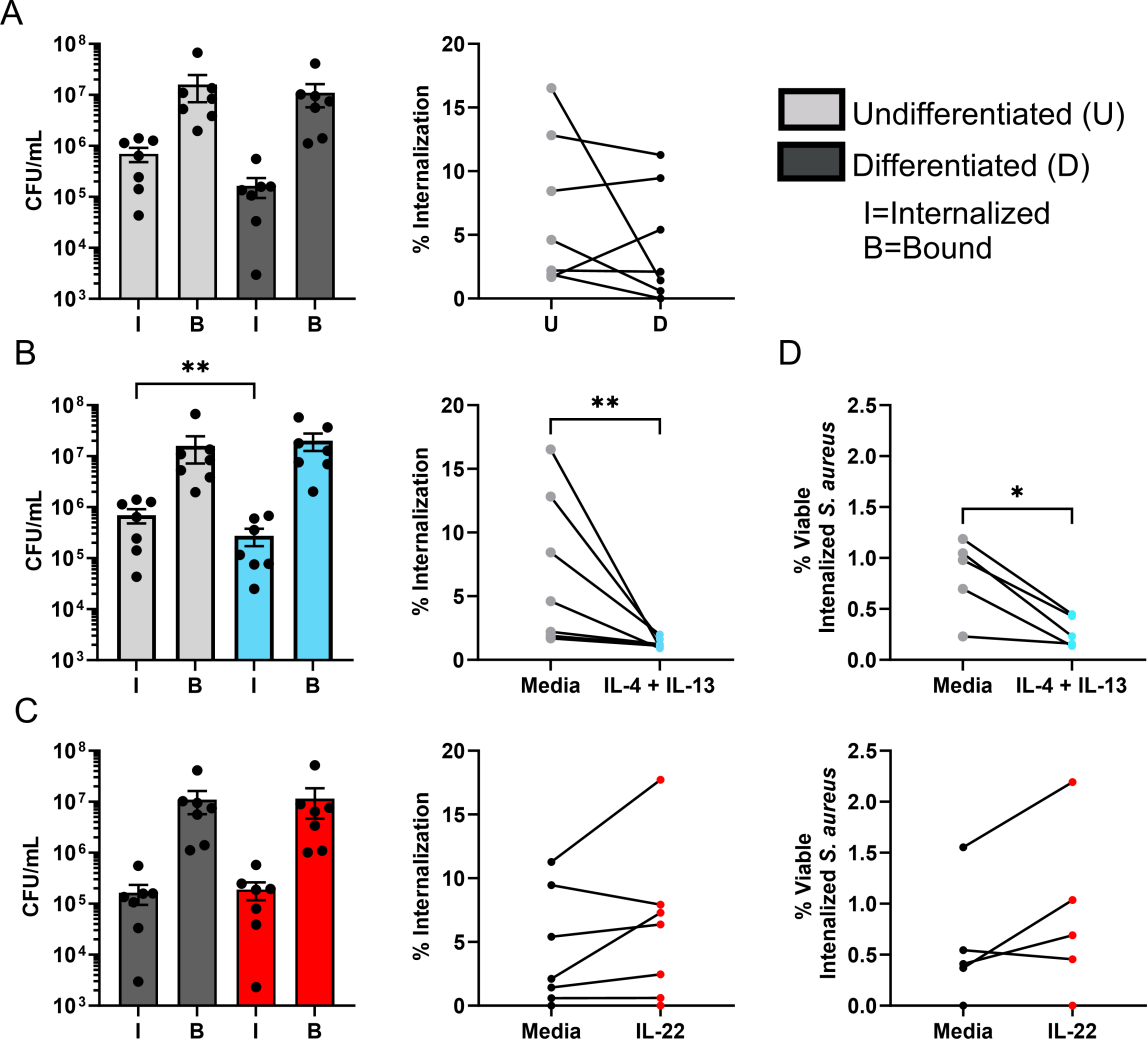
Experimental repeats with primary keratinocytes demonstrate similar results in genetically different cells. Primary human foreskin keratinocytes were exposed to an MOI of 100 *S*. *aureus* CFU per cell for 3 h. Extracellular bacteria were killed by gentamicin treatment and cells were lysed to quantify the number of bacteria internalized or bound represented by CFU/mL or the percent bacteria internalized (**A**). Bacterial internalization, binding, and the percent bacteria internalized with IL-4 + IL-13 (**B**) or IL-22 (**C**) cytokine treatment of primary keratinocytes at the relevant differentiation state. The percent viable internalized bacteria recovered from keratinocytes treated with either IL-4 + IL-13 or IL-22 and their respective controls (**D**). Results are expressed as a measurement of the fraction of live bacteria (culturable) to the total number of bacteria (live and dead measured from qPCR). Data are shown as mean ± SD. Statistical significance was tested with a paired nonparametric *t* test. *n* = 7 (**A–C**) or 5 (**D**) donors. Each line connects an individual donor. *P < 0.05, **P < 0.01.

To validate our key observations, these experiments were repeated in primary human foreskin keratinocytes (PHFKs). Similar to the results from immortalized keratinocytes, there was a reproducible decrease in bacterial internalization upon differentiation. However, the magnitude of this change varied substantially and did not always decrease with donors (Fig. 6A). Undifferentiated PHFK treated with IL-4 + IL-13 reaffirmed our observations of decreased *S. aureus* internalization (Fig. 6B) and treatment of differentiated PHFK with IL-22 also showed increased internalization across multiple (but not all) donors (Fig. 6C). Finally, the decrease in viable internalized *S. aureus* was observed in undifferentiated, IL-4 + IL-13-treated PHFK (Fig. 6D). No change in total internalized bacteria after cytokine treatment was detected by measuring *femA* values (Fig. S5B). Overall, our results from immortalized keratinocytes were recapitulated in keratinocytes from genetically different backgrounds.

## DISCUSSION

Understanding how keratinocyte differentiation and cutaneous inflammation affect *S. aureus* internalization into keratinocytes provides new insights on the interaction between the bacterium and skin. To study the importance of these characteristics of the skin we utilized both primary keratinocytes and the immortalized keratinocyte cell line N/TERT-2G. This particular immortalized cell line was chosen because we and others have shown them to form barriers and differentiate with similar kinetics and protein expression to primary cells (38, 39). Recently, it was shown in the immortalized keratinocyte cell line HaCaT that internalization of *S. aureus* was greater in strains isolated from AD subjects compared to laboratory strains (40). We previously showed that HaCaTs do not differentiate comparably to N/TERT-2G or primary cells by both protein expression of differentiation-associated markers and formation of barrier (39). While this paper provided an important precedent that disease strains of *S. aureus* are more readily internalized into keratinocytes than lab strains, it is difficult to understand what layer of the epidermis is being modeled by HaCaTs. Therefore, the importance of keratinocyte differentiation cannot be faithfully investigated in this cell line.

A key finding of this study is that differentiation diminishes *S. aureus* internalization into keratinocytes. This is in accordance with previous findings using HaCaT cells, which observed that less *S. aureus* was internalized into these cells following prolonged growth after confluency (an alternative method of keratinocyte differentiation) (41). Another important observation from these studies was that *S. epidermidis* is internalized into keratinocytes, albeit at a modest amount compared to *S. aureus*, and this was not influenced by differentiation. While limited *S. epidermidis* internalization into keratinocytes has been previously observed (7), we extended these observations by determining that keratinocyte differentiation did not influence this occurrence. A possible explanation for this difference in internalization between the two staphylococcal species is that *S. epidermidis* lacks a homolog of the fibronectin-binding protein (FnBP) (42, 43), which is thought to be critical for *S. aureus* internalization (34, 35). Undifferentiated keratinocytes are most reminiscent of the basal layer of the epidermis and acutely differentiated (24 h) keratinocytes, which we focused on in this study, more closely represent cells in the lower stratum spinosum. These findings suggest that the dominant target of *S. aureus* internalization is the lowest layer of the epidermis. Exposure to *S. aureus* most often occurs in the topmost layer (stratum corneum) of the epidermis in healthy skin (24, 25). Our results propose that individuals with skin barrier disruption (e.g., AD subjects) would be more likely to have greater internalized *S. aureus* since lower layers of the epidermis are more accessible. This could make AD subjects especially susceptible to: (i) infectious complications caused by *S. aureus* (staphylococcal scalded skin syndrome and impetigo), (ii) persistent colonization, (iii) chronic infections, and/or (iv) difficulty treating infections because of escape from antibiotic-mediated killing and the immune system (7, 44, 45). A future direction of the lab is to test this hypothesis by assessing the levels of culturable *S. aureus* in the epidermis using biopsies obtained from subjects with AD, subjects with AD who have recurrent infections with *S. aureus*, and healthy individuals.

To better understand why internalization of *S. aureus* decreased in differentiated keratinocytes, we measured the level of multiple surface receptors (*DSG1*, *HSPA8*, *HSPD1*, *ITGA5*, and *ITGB1*) reported to be important in binding/internalization (34). In support of these receptors changing during the process of differentiation, previous studies have indicated that *ITGA5* is primarily expressed by basal keratinocytes and decreases upon keratinocyte differentiation (46, 47). Of these receptors, α_5_ expression coincided the closest with *S. aureus* internalization, suggesting the other receptors may primarily mediate cell surface binding to keratinocytes. Ongoing research over the details of *S. aureus* interactions with skin cells have firmly established that *S. aureus* FnBPs are required for binding to keratinocytes to occur (48). This occurs through FnBPs binding to fibronectin, which is bound by α_5_β_1_ on the keratinocyte. Through this interaction, a bridge is created that allows *S. aureus* to interact with the skin cell (48, 49). Many of these studies have focused on HaCaTs, so understanding how the expression of host receptors that bind and mediate *S. aureus* internalization as a consequence of differentiation has not been studied thoroughly. Additionally, further investigation needs to be conducted to determine each receptor’s contribution to *S. aureus* internalization into keratinocytes, since the route of internalization is likely to be cell-dependent. This could be tested by using CRISPR/Cas9 to knock out each receptor and determine whether changes in internalization occur. Of note, although, α_5_ expression levels followed the same trend as *S. aureus* internalization in untreated conditions, it did not explain differences observed in cytokine-treated conditions. A possible reason why we saw a decrease in *ITGA5* mRNA after IL-4 + IL-13 treatment, but no change in protein levels, arises from previous work that demonstrated integrins have long degradation half-lives (12–24 h) in cells (50). Therefore, binding receptor expression cannot solely explain differences in bacterial internalization, suggesting an alternative mechanism(s) driving cytokine-mediated changes.

It is possible that individuals with chronic cutaneous diseases exhibiting increased susceptibility to bacterial infections have unique combinations of cytokines in the skin. To test this hypothesis, we used various disease-relevant cytokines indicative of either AD or psoriasis in our model (11, 13–15). In differentiated keratinocytes treated with IL-22, there was a modest increase in bacterial internalization. One possible explanation for this observation would be that IL-22, a cytokine known to be involved in keratinocyte proliferation, is delaying differentiation, allowing the cell to remain in a more undifferentiated state (51, 52). Since we have observed undifferentiated keratinocytes to internalize more *S. aureus* and there have been reports that indicate gene expression of keratinocytes treated with IL-22 appear to be more reminiscent of epidermal basal cells, this is a likely explanation of our results. Additionally, it is possible that retained expression of *ITGA5* may contribute to the increased internalization observed in IL-22-treated keratinocytes. This was noted by a reproducible increase in *ITGA5* mRNA (1.76 ± 0.74-fold) and protein (1.08 ± 0.05-fold) in IL-22 exposed cells compared to untreated controls. We also tested the effect of IL-4 + IL-13, on *S. aureus* internalization. Our initial hypothesis was that treatment with these AD-relevant cytokines would cause increased *S. aureus* internalization, which would be consistent with clinical observations of increased colonization/infections (4, 5). However, there was a significant decrease in bacterial internalization observed in undifferentiated keratinocytes treated with IL-4 + IL-13. Of note, a recent study demonstrated that HaCaT cells treated with IL-4 + IL-13 had no difference in internalized bacteria (40). A critical difference in this study was the focus on non-viable bacteria (heat-killed), while our study measured internalization of viable bacteria. Our measurement of total internalized bacteria through qPCR for *femA* concurred with their results since IL-4 + IL-13 did not change these values. To further investigate why IL-4 + IL-13-treatment diminished the number of culturable bacteria, we tested whether the viability of internalized *S. aureus* within cytokine-treated keratinocytes differed from untreated cells. Since previous literature has shown that intracellular *S. aureus* can be killed after being phagocytosed by macrophages (53), and recent reports have indicated keratinocytes are capable of killing intracellular *S. aureus* (31, 32), we hypothesized that IL-4 + IL-13 treatment was stimulating a keratinocyte-specific mechanism of killing internalized bacteria. By quantifying the total amount of bacterial genomes and culturable bacteria, we observed a lower percent of viable bacteria in keratinocytes treated with IL-4 + IL-13, suggesting a method of bacterial killing was being employed in cytokine-treated cells. However, what this keratinocyte-specific mechanism for eliminating internalized bacteria remains unknown, and is the focus of ongoing studies. It is important to note that the observations taken from these studies were not supportive of our original hypothesis that IL-4 + IL-13 would enhance bacterial internalization. To explain this discrepancy, we speculate that the increased number of keratinocytes containing dead bacteria promotes sustained and/or greater levels of inflammatory cytokine production. Since keratinocytes are known to produce a wide array of cytokines and chemokines in response to pathogens (54), it is possible that increased bacterial death (and by extension keratinocyte survival) contributes to the aberrant Th2 polarization observed in this disease. This would be testable by measuring the production of pro-Th2 cytokines (i.e., alarmins) from keratinocytes has internalized *S. aureus* and whether the amount or duration changes in the context of IL-4 + IL-13 treatment.

In this study, we demonstrated that the efficiency of *S. aureus* internalization depends on both the keratinocyte differentiation state and the epidermal cytokine environment. Understanding the characteristics that make keratinocytes more vulnerable to *S. aureus* internalization is important in understanding the pathogenesis of this bacterium. Further, this study suggests a potential mechanistic role for *ITGA5* in *S. aureus* internalization with regard to differentiation of keratinocytes. This has not been done due to previous studies mainly focusing on HaCaT cells as a model. By identifying characteristics of the skin that facilitate *S. aureus* internalization, it may be possible to identify individuals (such as individuals with chronic cutaneous diseases) at greater risk for infection.

## MATERIALS AND METHODS

### Cells and bacteria

The immortalized keratinocyte cell line N/TERT-2G and PHFKs were cultured as previously described (55, 56). Briefly, keratinocytes were maintained in keratinocyte serum-free media (KSFM) supplemented with epidermal growth factor and bovine pituitary extract (Cat #17005042, ThermoFisher Scientific). After plating, cells were grown to confluency and then differentiated for 24 h in DMEM (Cat #21068, ThermoFisher Scientific) supplemented with 1.8 mM CaCl_2_ and 4 mM L-glutamine, or left in KSFM, before exposure to bacteria. The USA300 (FRP3757; a derivative of the LAC strain) strain of *S. aureus*, and the HER1292 strain of *S. epidermidis*, were used for binding/internalization experiments (57, 58). Bacterial cultures were stored long-term in glycerol stocks at −80°C and freshly streaked onto tryptic soy agar (TSA) plates with 18 h of incubation to select single colonies for use in assays. TSA plates were made from tryptic soy broth (TSB) (Cat #211768, BD) and addition of agarose (Cat #214530, BD) as per company protocol.

### Growth curve

One colony of bacteria was inoculated into 5 mL of TSB and incubated at 37°C for 18 h. A 1:100 dilution was made of overnight culture in TSB. The culture tube was placed in an orbital shaker at 37°C and samples were taken at time 0 and then every hour for 7 h to quantify growth. Optical density was measured for each sample at a wavelength of 600 nm (OD_600_) using a SmartSpec Plus spectrophotometer (Bio-Rad, San Jose). A volume of 1 mL was set aside from each time point to be assessed for colony forming units per milliliter (CFU/mL) using 10-fold dilution increments, with 10 µL of each dilution spotted onto a TSA plate and then left for 18 h at 37°C to enumerate.

### Bacterial binding and internalization assays

One colony of bacteria was inoculated into 5 mL of TSB and incubated at 37°C for 18 h. A 1:10 dilution of this overnight culture was added to TSB and placed in an orbital shaker until grown to a sufficient concentration to provide 5E7 bacteria (MOI 100) per well. In a 24-well plate, cells (either N/TERT-2G or PHFK) were exposed to bacteria at an MOI of 100 for 3 h, washed three times with PBS, and subsequently treated with gentamicin (100 µg/mL) to kill extracellular bacteria (59). Gentamicin was added to all samples for internalization measurements while treatment groups used to determine binding did not receive the antibiotic. All wells were washed three times with PBS, then PBS containing 0.1% Triton X-100 was added to lyse keratinocytes and release internalized bacteria. To enumerate CFU/mL, the content of each well was collected into Eppendorf tubes and spotted onto TSA plates after dilution and then left at 37°C for 18 h. Since binding conditions included all bacteria that were internalized and bound, binding was calculated by subtracting the number of internalized bacteria from the number of bacteria in the binding condition.

### LDH release assay

An LDH Release assay kit (Cat #04744926001, Roche) was used to determine cellular viability after exposure to gentamicin. Cells were plated in a 96-well, flat bottom plate, grown to confluency, and then treated with gentamicin for the respective amounts of time used in internalization and binding assays. A positive control was generated by the treatment of cells with 5 µL of the provided lysis buffer for 15 min at 37°C. About 50 µL of cell supernatant was then removed from cells and added to a separate 96-well, flat-bottom plate and 50 µL of the catalyst and dye mixture (1:45 dilution of catalyst to dye) was added to all wells. The plate was incubated for 10 min at 37°C and the reaction was stopped by adding 25 µL of stop solution to each well. Absorbance was measured at 490 nm and 620 nm (to measure background) on a SpectraMaxi3x plate reader (Molecular Devices). 620 nm values were subtracted from 490 nm values to remove background signal and then a media only (no cells) condition was subtracted from all conditions. Viability was calculated by taking the ratio of sample to lysed control and multiplying by 100 to obtain % viability.

### qPCR

#### mRNA isolation

Keratinocytes at various stages of differentiation, either cultured in KSFM (undifferentiated) or DMEM containing high calcium (1.8 mM) for 24 h (differentiated), were harvested by washing each well with 500 µL PBS and then resuspending in 250 µL of tri-reagent (Cat #93289, ThermoFisher Scientific) for collection. RNA was isolated using an mRNA isolation kit (Cat #R683402, Omega Biotek) via the vendor-supplied protocol.

#### cDNA synthesis

All samples were quantified for mRNA (ng/µL) using a NanoDrop Spectrophotometer (ThermoFisher Scientific). About 300 ng of mRNA was used per sample. DEPC water was added to each sample to reach an equal volume for all samples (15 µL). The mRNA/DEPC mixture was added to 4 µL of 5× buffer and 1 µL of polymerase (Cat #170-8891, Bio-Rad) for a total reaction volume of 20 µL. Using a thermal cycler (ThermoFisher Scientific), samples were run for 5 min at 22°C, 30 min at 42°C, and finally 5 min at 85°C.

#### Quantification of Gene Expression

In a 96-well plate, each well received 4 µL of cDNA mix (0.4 µL of cDNA and 3.6 µL of DEPC water) and 6 µL of primer mix [5 µL SYBR (Cat #95053-02K, Quantabio) and 1 µL primer (10 µM stock)]. The plate was then sealed with an adhesive lid, placed in a Bio-Rad CFX Connect qPCR machine, and run with the following protocol: initial denaturation of 94°C for 3 min, followed by 39 cycles of 15 s at 94°C and then 1 min at 55°C. Primers for genes of interest were ordered through Integrated DNA Technologies (Table 1). Human *GAPDH* was used as a housekeeping gene.

**TABLE 1:** Primers used

Gene name	Forward (5′-3′)	Reverse (5′-3′)
*DSG1*	TCAATCCGAAGGCAGAAACG	TGCGGTATGTAACTTGCTGG
*GAPDH*	ACATCGCTCAGACACCATG	TGTAGTTGAGGTCAATGAAGGG
*HSPA8*	CACTTGGGTGGAGAAGATTTTG	CTGATGTCCTTCTTATGCTTGC
*HSPD1*	GTCTTCAGGTTGTGGCAGTC	GGCATCGTCTTTGGTCACAA
*ITGA5*	TCATCTACATCCTCTACAAGCTTGG	GCCGTCAGCACCTTCAAGA
*ITGB1*	GAAGGGTTGCCCTCCAGA	GCTTGAGCTTCTCTGCTGTT

### Flow cytometry

Plates were prepared using the same method as those for qPCR analysis. N/TERT2G cells at different stages of differentiation (undifferentiated or 1-day post-differentiation) were harvested with 100 µL of TrypLE Express in each well. Contents were transferred into separate Eppendorf tubes and centrifuged at 1,250 rpm for 5 min to pellet cells. The supernatant was aspirated and 500 µL of FACS buffer (PBS with 3% fetal bovine serum albumin and 1 mM EDTA) was used to wash the cells two times. Samples were resuspended in 50 µL of a 1:50 dilution of Human TruStain FcX (Cat #422301, BioLegend) and left on ice for 20 min. Then 50 µL of a 1:100 dilution of the ITGA5 monoclonal primary antibody (Cat #14-0496-82, Invitrogen/eBioscience) was added, and samples were left on ice for 60 min. Next, the samples were washed two times with FACS buffer before the addition of a 1:500 dilution of Alexa 488 (anti-mouse) secondary antibody (Cat #A21202, Invitrogen). Samples were then left on ice for 60 min, after which three washes were performed and everything was resuspended in 200 µL of FACS buffer. Samples were analyzed with an AccuriC6 flow cytometer (BD Biosciences) and results were analyzed with FlowJo software version 10.8.1.

### Bacterial genomic qPCR

#### Standard curve preparation

One colony of bacteria was inoculated into 5 mL of TSB and incubated at 37°C for 18 h. A 1:10 dilution of this overnight culture was added to TSB and placed in an incubating orbital shaker until desired growth was reached by OD_600_ readings (number of bacteria in the culture was determined from previous growth curve experiments performed). 5E8 bacteria were isolated from culture and spun down at 4,000 × *g* for 10 min. The supernatant was removed and the bacteria were resuspended in 500 µL of DNA/RNA shield. Samples were placed on a bead ruptor (Omni International) at medium speed for 60 s, and this was repeated a total of three times. The ZymoBIOMICS DNA Miniprep kit (Cat. #D4304, Zymo Research) was then used with the vendor-supplied protocol. In the final step, gDNA was eluted in 50 µL DEPC water. To generate the standard curve, isolated gDNA copies (10^7^, 10^6^, 10^5^, 10^4^, 10^3^, and 10^2^) were used. Each well received 5 µL SYBR, 3 µL DEPC water, 1 µL *femA* primer set (F: 5′-AACTGTTGGCCACTATGAGT-3′, R: 5′-CCAGCATTACCTGTAATCTCG-3′), and 1 µL of diluted gDNA. The plate was then sealed with an adhesive lid and placed in a BioRad CFX Connect qPCR machine and run with the following protocol: initial denaturation of 94°C for 10 min, followed by 39 cycles of 15 s at 94°C and then 1 min at 55°C.

#### Isolating bacterial gDNA and femA qPCR

Samples from the *S. aureus* binding/internalization assays were spun down at 7,000 rpm for 10 min, and the same gDNA isolation protocol was followed as described above. The standard curve and samples each were added to the same reaction mixture: 5 µL SYBR, 3 µL DEPC water, 1 µL *femA* primer set, and 1 µL of diluted gDNA. The plate was then sealed with an adhesive lid and placed in a BioRad CFX Connect qPCR machine. Each sample was compared back to the standard curve to determine the total quantity of bacteria.

## SUPPLEMENTAL MATERIAL

Supplemental Material msphere.00685-23-s0001.pdf Fig. S1-S5.

ASM does not own the copyrights to Supplemental Material that may be linked to, or accessed through, an article. The authors have granted ASM a non-exclusive, world-wide license to publish the Supplemental Material files. Please contact the corresponding author directly for reuse.
