# Supplementary material for: Differentiation of keratinocytes or exposure to type 2 cytokines diminishes S. aureus internalization
Source: mSphere. 2024 Mar 19;9(4):e00685-23. doi: 10.1128/msphere.00685-23 (PMC11036805; doi:10.1128/msphere.00685-23)
Supplement: Supplemental Material — Fig. S1-S5. [file msphere.00685-23-s0001.pdf]

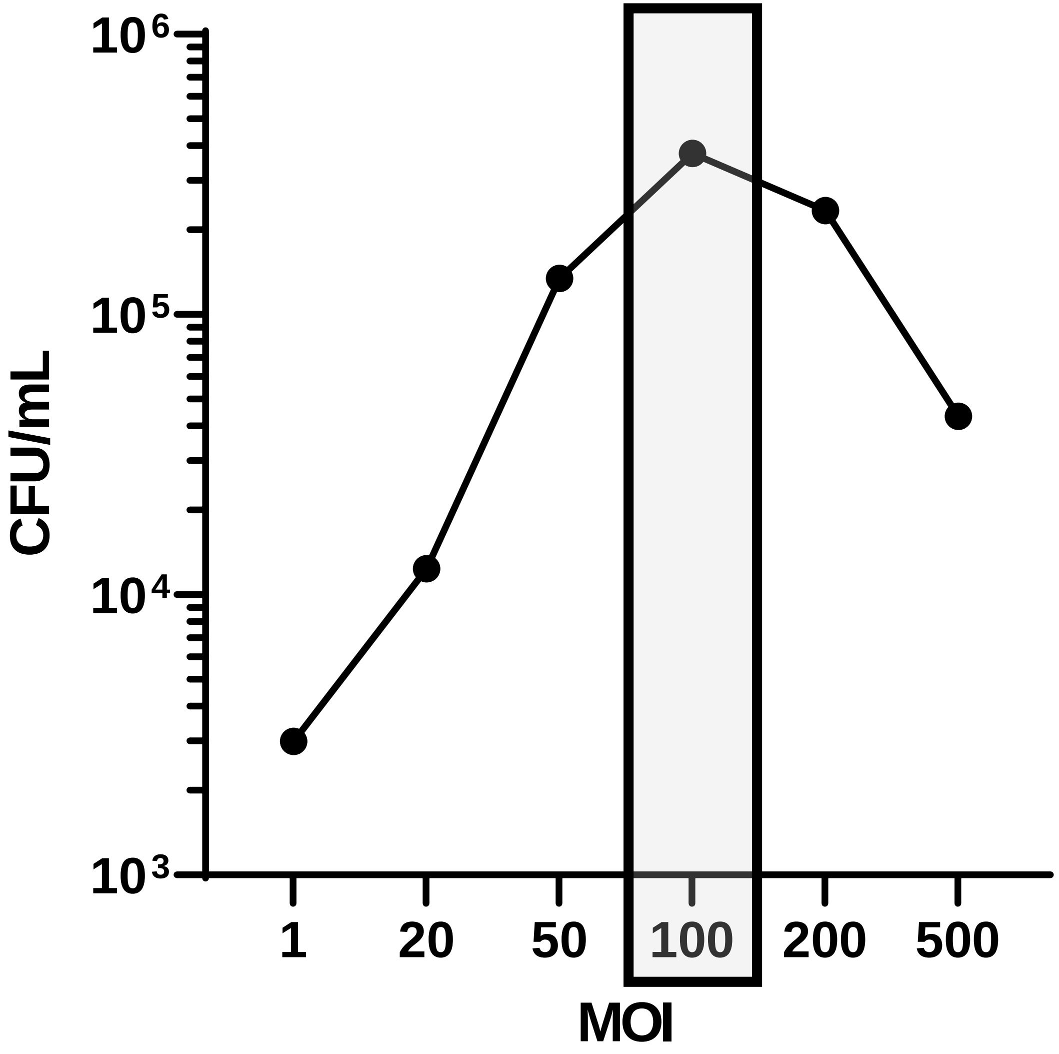

Supplementary Figure 1. Multiplicity of infection (MOI) of 100 was identified as the ideal amount of bacteria to use in *S. aureus* binding/internalization assays. N/TERT-2G cells were infected for 3 hours with *S. aureus* at MOIs ranging from 1 to 500. The cells were then treated with gentamicin for 1 hour to kill extracellular bacteria and subsequently lysed with 0.1% Triton X-100 in PBS. Lysates were plated onto tryptic soy agar plates, incubated for 18 hours, and bacterial colonies were enumerated. The data points are the average of n = 2 experiments. CFU/mL, colony forming units per milliliter

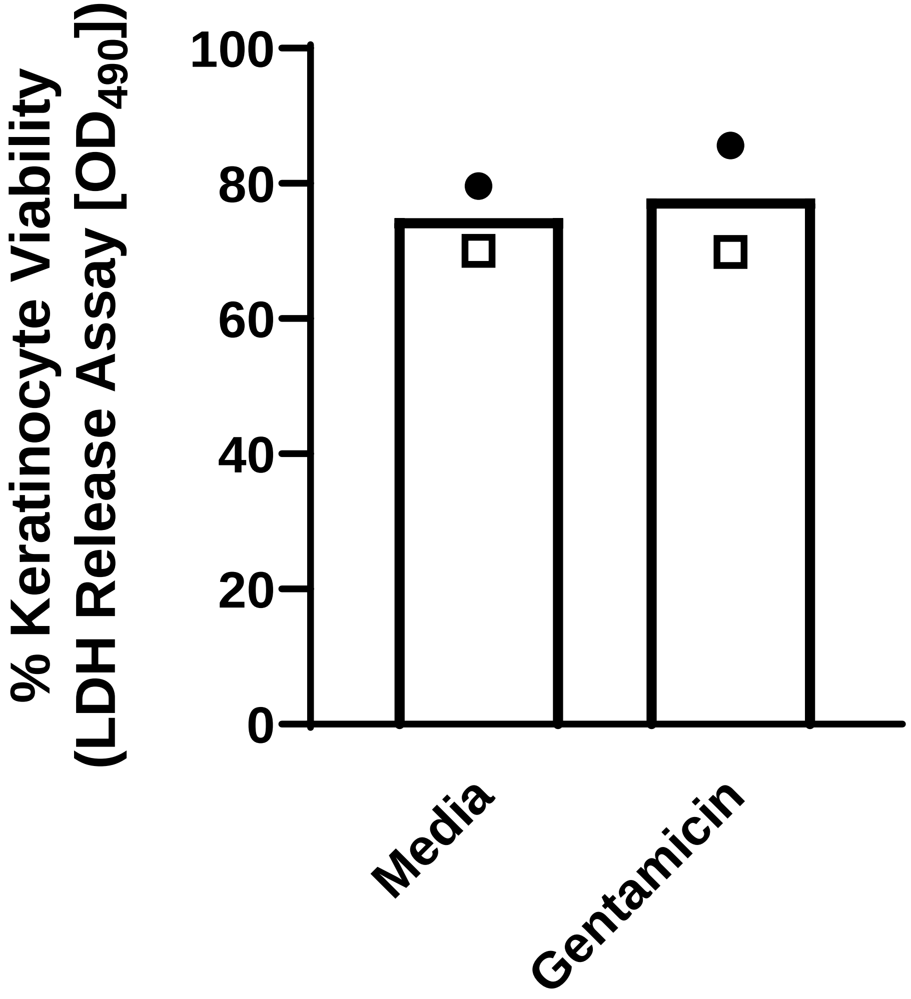

Supplementary Figure 2. Treatment with gentamicin does not impact keratinocyte viability. N/TERT-2G were left untreated or treated with gentamicin at 100 µg/ml for 1 hour. Supernatants were removed from cells and tested for cell death with an LDH release assay. Each symbol represents an individual experiment. n = 2 experiments

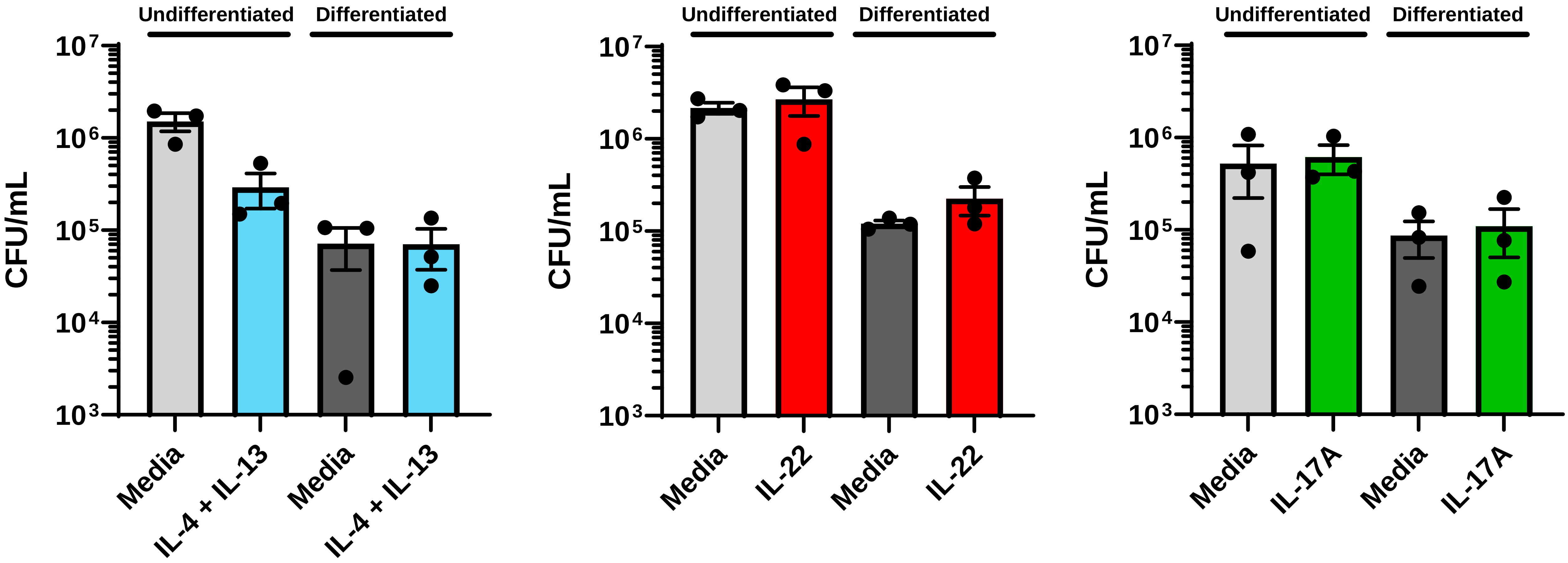

Supplementary Figure 3. *S. aureus* internalization is only reduced in undifferentiated keratinocytes treated with IL-4 + IL-13 and increased in differentiated keratinocytes treated with IL-22. Undifferentiated or differentiated N/TERT2-G cells were treated for 24 hours with IL-4 + IL-13, IL-22 or IL-17A (50 ng/ml) and then exposed to a MOI of 100 *S. aureus* CFU per cell for 3 hours. Extracellular bacteria were killed by gentamicin treatment and cells were lysed to quantify the number of internalized bacteria. Data is shown as mean ± SEM. n = 3 experiments

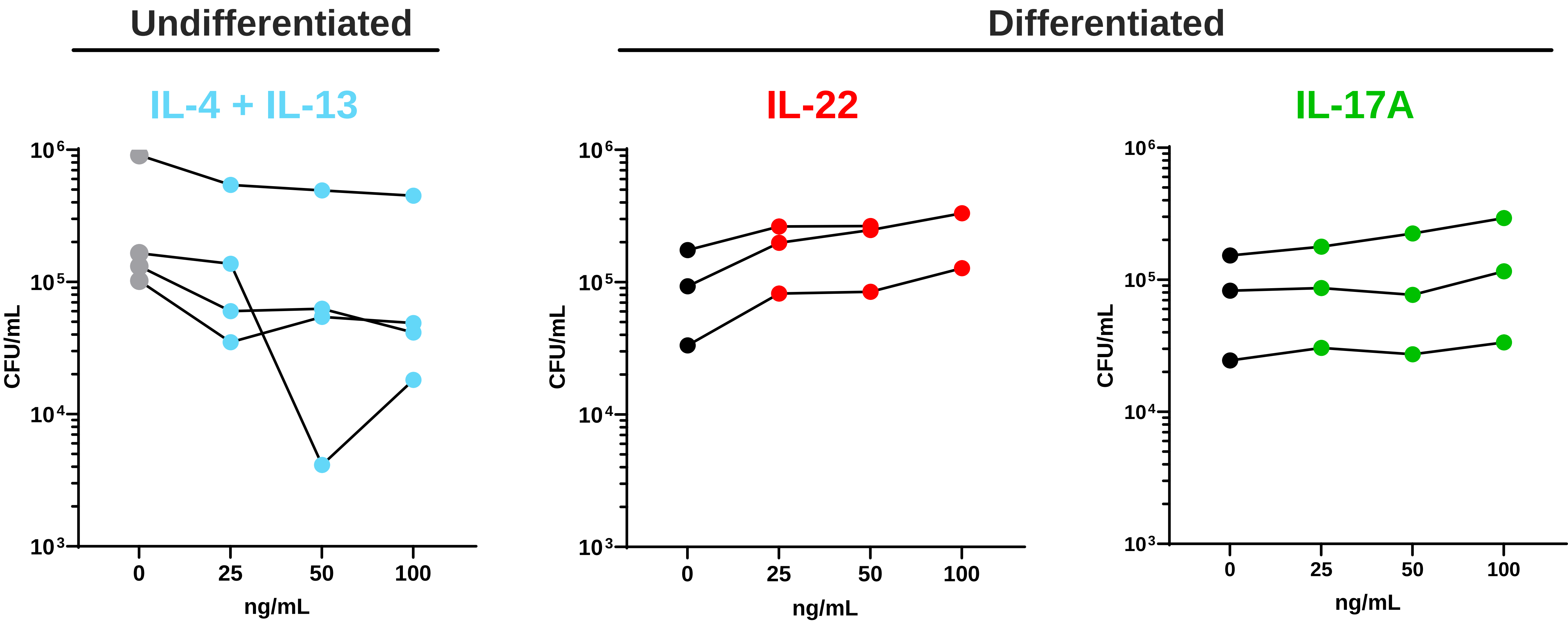

Supplementary Figure 4. Dose response curves for the cytokines used in our studies. Undifferentiated and differentiated keratinocytes were left untreated or treated with either IL-4 + IL-13, IL-22, or IL-17A cytokines at various doses (25, 50, or 100 ng/mL) and then assessed for *S. aureus* internalization. The number of internalized *S. aureus* is displayed in CFU/mL. n = 3 (IL-22, IL-17A) or 4 (IL-4 + IL-13) experiments. Each line connects an individual experiment.

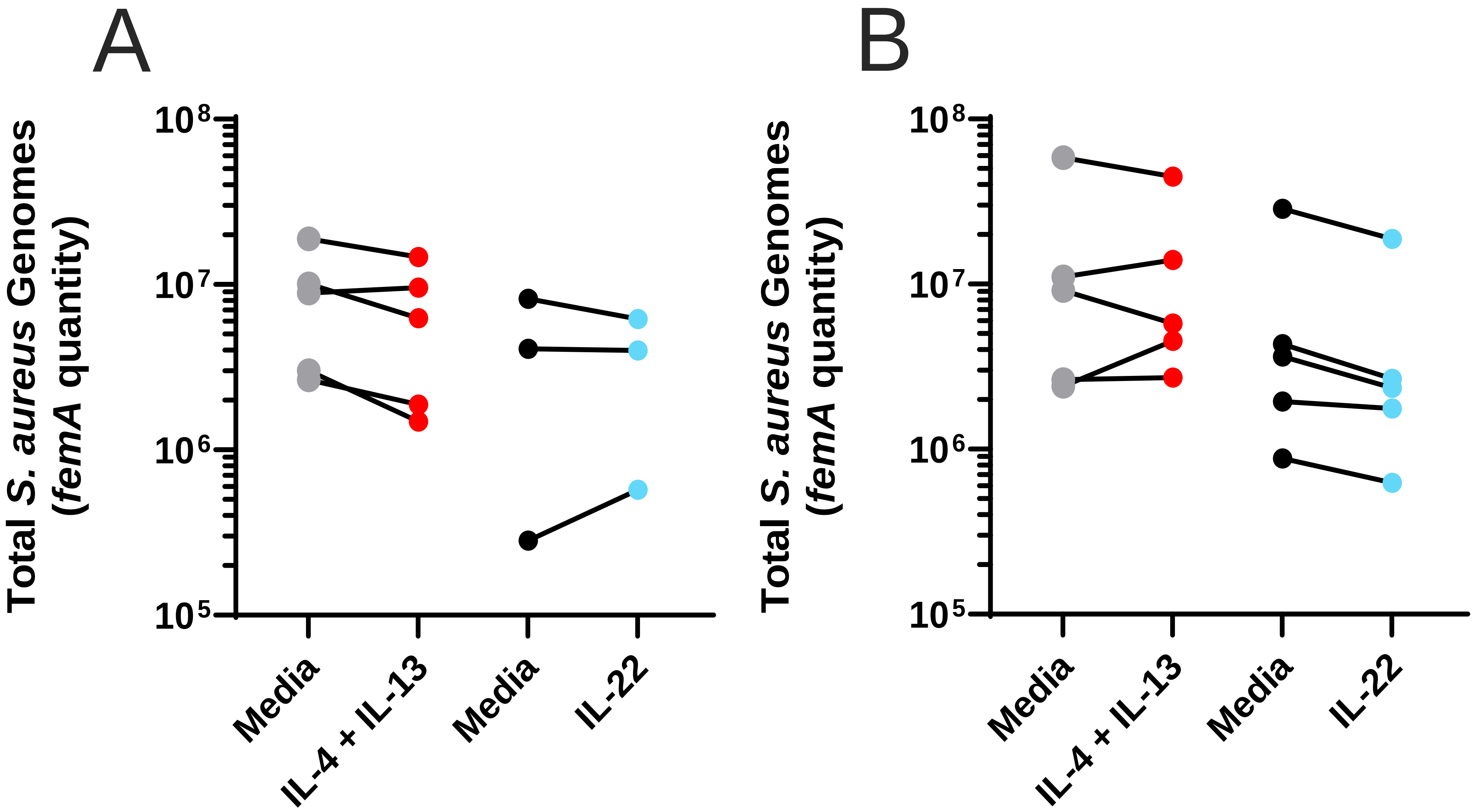

Supplementary Figure 5. The total amount of bacterial genomes does not differ in keratinocytes undergoing differentiation or treated with cytokines. gDNA was harvested from keratinocytes with internalized *S. aureus* and the total amount of *S aureus* in each sample was quantified through qPCR for the *femA* gene using a standard curve derived from a known number of bacteria. *femA* quantity from N/TERT-2G cells (Figure 3, **A**) or primary keratinocytes (Figure 6, **B**). n = 3-5 experiments.
